# Body fatness and mTOR pathway activation of breast cancer in the Women’s Circle of Health Study

**DOI:** 10.1038/s41523-020-00187-4

**Published:** 2020-09-21

**Authors:** Ting-Yuan David Cheng, Angela R. Omilian, Song Yao, Pamela V. Sanchez, Latasia Z. Polk, Weizhou Zhang, Susmita Datta, Wiam Bshara, Rochelle Payne Ondracek, Warren Davis, Song Liu, Chi-Chen Hong, Elisa V. Bandera, Thaer Khoury, Christine B. Ambrosone

**Affiliations:** 1 Department of Epidemiology, University of Florida, Gainesville, FL USA; 2 Department of Cancer Prevention and Control, Roswell Park Comprehensive Cancer Center, Buffalo, NY USA; 3 Department of Pathology, Immunology and Laboratory Medicine, University of Florida, Gainesville, FL USA; 4 Department of Biostatistics, University of Florida, Gainesville, FL USA; 5 Department of Pathology & Laboratory Medicine, Roswell Park Comprehensive Cancer Center, Buffalo, NY USA; 6 Department of Biostatistics and Bioinformatics, Roswell Park Comprehensive Cancer Center, Buffalo, NY USA; 7 Cancer Epidemiology and Health Outcomes, Rutgers Cancer Institute of New Jersey, The State University of New Jersey, New Brunswick, NJ USA

**Keywords:** Cancer epidemiology, Risk factors, Biomarkers

## Abstract

Energy imbalance has an important role in breast cancer prognosis. Hyperactive mechanistic Target of Rapamycin (mTOR) pathway is associated with breast tumor growth, but the extent to which body fatness is associated with mTOR pathway activities in breast cancer is unclear. We performed immunostaining for mTOR, phosphorylated (p)-mTOR, p-AKT, and p-p70S6K in tumor tissue from 590 women (464 African Americans/Blacks and 126 Whites) with newly diagnosed invasive breast cancer in the Women’s Circle of Health Study. Anthropometric measures were taken by study staff, and body composition was measured by bioelectrical impedance analysis. Linear regressions were used to estimate percent differences in protein expression between categories of body mass index (BMI), waist circumference, waist/hip ratio, fat mass, fat mass index, and percent body fat. We observed that BMI ≥ 35.0 vs. <25 kg/m^2^ was associated with 108.3% (95% CI = 16.9%–270.9%) and 101.8% (95% CI = 17.0%–248.8%) higher expression in p-mTOR and normalized p-mTOR, i.e., p-mTOR/mTOR, respectively. Quartiles 4 vs. 1 of waist/hip ratio was associated with 41.8% (95% CI = 5.81%–89.9%) higher mTOR expression. Similar associations were observed for the body fat measurements, particularly in patients with estrogen receptor-negative (ER−) tumors, but not in those with ER+ tumors, although the differences in associations were not significant. This tumor-based study found positive associations between body fatness and mTOR pathway activation, evident by a p-mTOR expression, in breast cancer. Our findings suggest that mTOR inhibition can be a treatment strategy to prevent the recurrence of these tumors in obese individuals.

## Introduction

Energy imbalance has an important role in breast cancer prognosis: compared with individuals with normal weight (18.5–24.99 kg/m^2^) at diagnosis, those with obesity (body mass index [BMI] ≥ 30 kg/m^2^) are associated with a 30% increased risk of all-cause or breast cancer-specific mortality, regardless of menopausal status or tumor estrogen receptor (ER) status^1–3^. The aromatization process in adipose tissue produces estrogens that promote ER+ cancer; however, the hormonal pathway cannot fully account for the association because the association is also observed in patients who receive tamoxifen or aromatase inhibitors^4,5^ and in patients with ER− tumors^1^. In addition, although the evidence is less compared to BMI, central adiposity is associated with poor prognosis in patients with breast cancer^6^, and the mechanism between central adiposity and outcomes may be different from that of BMI and outcome. Although some clinical trials examining behavioral changes (e.g., weight reduction through a decreased fat intake and increased physical activity) reveal promising findings^7,8^, it is crucial to identify mechanisms through which overall and central adiposity exert their effects. Lifestyle interventions may not be applicable or effective for all women with breast cancer; targeting the underlying biological mechanisms may open new opportunities to improve the prognosis for a greater number of patients.

**Fig. 1:**
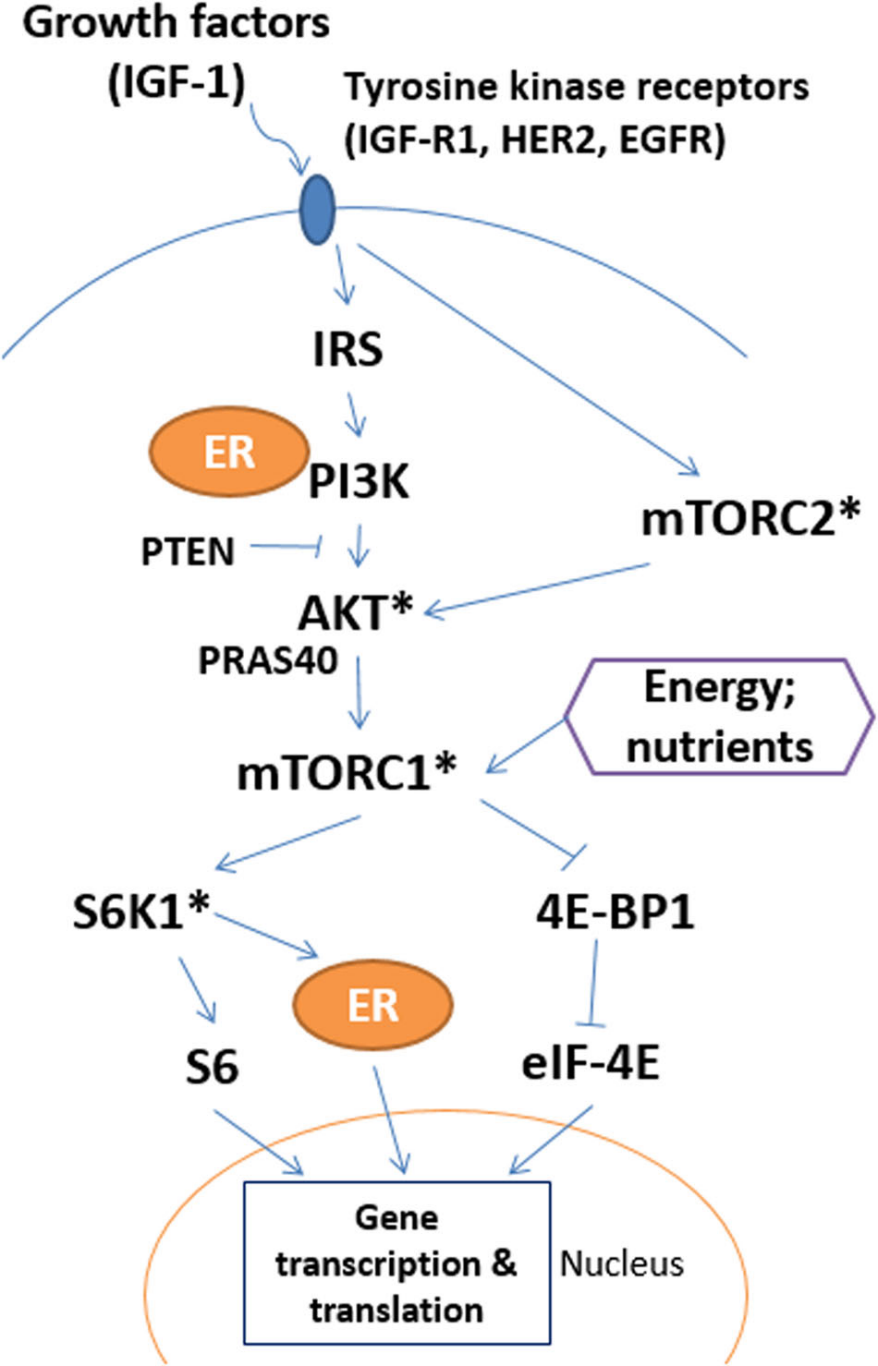
Putative mechanism of the mTOR pathway. *Protein expression was assessed using IHC in this study. 4E-BP1 4E-binding protein-1, AKT Protein kinase B, eIF-4E eukaryotic initiation factor-4E, IRS insulin receptor substrate, mTORC1, and mTORC2 mTOR Complex 1 and 2, PI3K phosphoinositide 3-kinases, PRAS40 proline-rich Akt substrate 40 kDa, PTEN phosphatase and tensin homolog, S6K1 S6 kinase-1 (also known as p70S6).

An obesity-related signaling pathway is a mechanistic Target of Rapamycin (mTOR) pathway (Fig. 1). The mTOR pathway is activated by energy influx, amino acids, and insulin-like growth factors (IGFs)^9^, and activation of the pathway promotes several cancer hallmarks, such as cell proliferation and angiogenesis^10,11^. and is associated with an increased risk of breast cancer recurrence^12^. The main signaling mechanism of the pathway is protein phosphorylation, a post-translational process^11^. Phosphorylated mTOR and its upstream and downstream proteins, such as p70S6 kinase (p70S6K), are highly expressed in breast tumors^13–16^. However, data are very limited on the association between obesity phenotypes and mTOR pathway activation in breast tumors. This knowledge would advance our understanding of the mechanism of how energy imbalance affects breast cancer prognosis and shed light on the potential for promoting energy balance and mTOR inhibition as strategies to improve clinical outcomes^17,18^.

Here, we investigated the mTOR pathway activities in breast tumors in association with body size, i.e., BMI, waist circumference (WC), and waist/hip ratio (WHR), as well as body composition, i.e., fat mass, fat mass index, and percent body fat. We hypothesized that mTOR pathway activities would be higher in women with larger body size and more fat mass, compared to those with smaller body size and less fat mass. Because the influence of obesity may differ between the ER+ and ER− subtypes of breast cancer^19,20^, we also examined the mTOR-body fatness associations stratified by ER status.

## Results

### Protein expression and patient characteristics

The distributions of *H*-scores for IHC markers are listed in Table 1. mTOR and p-mTOR expression levels were modestly correlated (*r* = 0.35; Supplementary Table 1). The expression levels of phosphoproteins, i.e., p-mTOR, p-AKT, and p-p70S6K were also modestly correlated with each other (*r* = 0.27–0.54). Table 2 shows the univariate analysis of the protein expression levels of mTOR, p-mTOR, normalized p-mTOR, and total phosphoproteins according to demographic and clinicopathological characteristics. mTOR expression was higher in tumors from Black than White women (median *H*-score = 145 vs. 96, *P* < 0.001). Patients with a tumor of lower grade and smaller size and an earlier stage of breast cancer had higher expression levels of p-mTOR and total phosphoproteins than those with a tumor of higher grade and larger size and a later stage of the disease. The expression levels of mTOR, p-mTOR, and total phosphoproteins were higher in ER+ and PR+ than ER− and PR− tumors; the expression levels were lowest in triple-negative tumors among the molecular subtypes. In multivariable models, the Black race compared to the White race was significantly associated with higher expression of mTOR and p-mTOR, but not normalized p-mTOR or total phosphoproteins, in breast tumors after adjusting for age, menopausal status, history of diabetes, grade, stage, molecular subtype, and BMI category (Supplementary Table 2).

**Table 1:** Distributions of *H*-scores for the IHC markers (*N* = 590).

IHC marker	Mean	SD	Median	Interquartile range
mTOR^a^	133	72	135	79–189
p-mTOR^a^	47	58	19	1–83
p-AKT^b^	41	63	9	0–58
p-p70S6K^b^	54	80	10	0–83
Total phosphoprotein^c^	143	141	102	20–224
Normalized p-mTOR^d^	176	2488	16	2–58

^a^Scored in the cytoplasm.

^b^Summation of *H*-scores in the cytoplasm and in nuclei.

^c^Summation of *H*-scores of p-mTOR, p-AKT, and p-p70S6K.

^d^Normalized p-mTOR was calculated as the *H*-score of p-mTOR divided by the *H*-score of mTOR multiplied by 100.

**Table 2:** Protein expression of the mTOR pathway in breast cancer according to demographic and clinicopathological characteristics (*N* = 590).

	*N*	mTOR	p-mTOR	Normalized p-mTOR	Total phosphoproteins
Race					
Black	464	145 (88–199)	20 (2–84)	16 (2–54)	106 (20–228)
White	126	96 (52–141)	15 (1–75)	17 (2–86)	87 (23–209)
* P*-value^b^		<0.001	0.39	0.96	0.23
Age					
<40	74	140 (75–199)	21 (1–72)	14 (2–49)	92 (20–271)
40–49	185	127 (80–183)	16 (2–81)	16 (2–58)	99 (18–203)
50–59	263	132 (79–187)	19 (2–85)	17 (2–60)	103 (24–222)
≥60	68	148 (80–207)	25 (1–96)	16 (1–50)	137 (18–261)
* P*-value		0.43	0.68	0.61	0.46
Menopausal status					
Premenopausal	275	128 (78–187)	19 (2–81)	16 (2–55)	99 (20–216)
Postmenopausal	315	139 (79–190)	20 (1–85)	16 (2–58)	107 (20–230)
* P*-value		0.49	0.67	0.32	0.97
History of diabetes					
Ever^a^	78	140 (97–195)	26 (1–85)	15 (1–46)	114 (16–219)
Never	512	131 (76–187)	18 (2–83)	16 (2–58)	101 (20–225)
* P*-value		0.16	0.68	0.72	0.77
Tumor grade					
Low	80	142 (87–202)	56 (11–121)	45 (12–78)	126 (50–260)
Intermediate	203	136 (87–191)	43 (9–100)	34 (9–73)	135 (48–239)
High	288	131 (76–184)	7 (1–45)	7 (1–33)	70 (7–193)
* P*-value		0.32	<0.001	0.27	<0.001
Tumor size (cm)					
<1.0	75	147 (83–219)	42 (9–114)	34 (9–63)	160 (53–291)
1.0–1.9	218	128 (80–185)	18 (2–86)	18 (3–58)	103 (17–226)
≥2.0	283	134 (76–187)	15 (1–72)	13 (1–54)	84 (20–208)
* P*-value		0.21	0.009	0.63	0.006
AJCC stage					
I	256	139 (80–197)	27 (3–89)	22 (4–63)	127 (46–238)
II	242	133 (79–187)	15 (1–77)	13 (1–55)	83 (20–206)
III, IV	86	125 (71–169)	14 (1–49)	11 (2–47)	75 (7–209)
* P*-value		0.15	0.011	0.09	0.022
Lymph node status					
Negative	307	139 (80–194)	21 (2–84)	16 (2–58)	107 (17–226)
Positive	216	129 (76–181)	18 (1–68)	15 (2–57)	85 (21–219)
* P*-value		0.12	0.37	0.21	0.47
ER status					
Positive	396	140 (87–197)	44 (7–100)	32 (7–69)	138 (43–259)
Negative	194	119 (67–172)	3 (0–20)	3 (0–21)	47 (3–135)
* P*-value		0.002	<0.001	0.36	<0.001
PR status					
Positive	374	143 (87–198)	43 (6–103)	31 (7–71)	129 (34–248)
Negative	214	115 (71–172)	4 (0–23)	5 (1–25)	58 (5–184)
* P*-value		0.002	<0.001	0.33	<0.001
HER2 status					
Positive, equivocal	108	129 (78–173)	17 (2–47)	13 (2–38)	104 (29–250)
Negative	478	136 (77–191)	21 (1–87)	18 (2–58)	100 (17–219)
* P*-value		0.49	0.07	0.70	0.20
Molecular subtype					
HR+/HER2−	352	139 (84–196)	45 (6–103)	34 (7–70)	129 (34–237)
HR+/HER2+	68	140 (87–191)	18 (3–66)	14 (3–46)	154 (36–298)
HR−/HER2+	40	109 (69–142)	10 (2–31)	10 (2–26)	66 (20–178)
HR−/HER2−	126	119 (67–175)	2 (0–12)	2 (0–13)	28 (3–116)
* P*-value		0.045	<0.001	0.79	<0.001

Values are median (interquartile range) of *H*-score.

^a^Any self-report history or medication use of diabetes.

^b^ANOVA.

### Body fatness measurements

Among the body fatness measurements, BMI was highly correlated with WC (*r* = 0.88), fat mass (*r* = 0.93), fat mass index (*r* = 0.97), and percent body fat (*r* = 0.80) (Supplementary Table 3). WHR was modestly correlated with WC (*r* = 0.57), and BMI (*r* = 0.34). The correlations were high (*r* = 0.91–0.98) among the fat mass, fat mass index, and percent body fat.

### Body fatness and protein expression

BMI was positively associated with greater expression of p-mTOR and normalized p-mTOR (*P*-trend = 0.011 and 0.046, respectively; Table 3). Tumors from women with BMI ≥ 35 vs. those with BMI < 25 kg/m^2^ had 108.3% (95% CI = 16.9%–270.9%, *P* = 0.012) higher level of p-mTOR expression and 101.8% (95% CI = 17.0%–248.8%, *P* = 0.012) higher level of normalized p-mTOR expression. Quartiles 4 vs. 1 of WHR was associated with 41.8% (95% CI = 5.81%–89.9%, *P* = 0.019, *P*-trend = 0.023) higher level of mTOR expression. For body composition measurements (Table 4), higher vs. lower fat mass, fat mass index, and percent body fat were associated with greater p-mTOR and normalize p-mTOR expression (all *P*-trend <0.05). Quartiles 4 vs. 1 of fat mass, fat mass index, and percent body fat were associated with 149.8%, 124.1%, and 123.9% higher levels of p-mTOR expression, respectively (all *P* < 0.05). The estimates were similar for normalized p-mTOR expression. We observed a lower mTOR expression among women with overweight (vs. normal weight) and those in the second quartile of percent body fat (vs. the first quartile). However, these associations were not observed for the phosphoproteins. There was no association of total phosphoprotein, p-AKT, or p-p70S6K (Supplementary Table 4) with the body fatness measurements.

**Table 3:** Associations between body size and mTOR pathway activities.

	*N*	mTOR		p-mTOR		Normalized p-mTOR		Total phosphoprotein	
		Percent difference (95% CI)^a^	*P*-value	Percent difference (95% CI)^a^	*P*-value	Percent difference (95% CI)^a^	*P*-value	Percent difference (95% CI)^a^	*P*-value
BMI, kg/m^**2**^									
<25	131	Ref.		Ref.		Ref.		Ref.	
25–29.99	164	−28.0 (−45.1, −5.5)	0.018	12.4 (−35.0, 94.3)	0.67	55.8 (−7.1, 161.2)	0.09	−14.3 (−46.2, 36.5)	0.52
30–34.99	146	6.3 (−19.7, 40.7)	0.67	23.7 (−29.7, 117.7)	0.46	16.5 (−31.7, 98.7)	0.57	−38.2 (−59.5, −5.6)	0.023
≥35	144	3.2 (−22.5, 37.5)	0.82	108.3 (16.9, 270.9)	0.012	101.8 (17.0, 248.2)	0.012	−5.4 (−38.6, 45.8)	0.80
* P*-trend		0.19		0.011		0.046		0.17	
WC, cm									
Q1 (≤87.90)	151	Ref.		Ref.		Ref.		Ref.	
Q2 (87.91–98.40)	144	11.2 (−14.9, 45.3)	0.44	59.4 (−7.2, 173.9)	0.09	43.5 (−14.0, 139.4)	0.17	−6.6 (−41.2, 48.4)	0.77
Q3 (98.41–110.00)	143	33.1 (1.51, 74.5)	0.039	62.7 (−5.8, 181.2)	0.08	22.5 (−27.0, 105.4)	0.44	−9.7 (−42.5, 42.1)	0.66
Q4 (>110.00)	137	18.2 (−11.0, 57.0)	0.25	66.4 (−9.6, 184.8)	0.11	36.0 (−20.9, 133.9)	0.27	0.35 (−35.8, 56.9)	0.98
* P*-trend		0.12		0.11		0.38		0.68	
WHR									
Q1 (≤0.82)	150	Ref.		Ref.		Ref.		Ref.	
Q2 (0.83–0.88)	147	31.7 (0.80, 72.1)	0.044	24.9 (−27.4, 115.0)	0.42	−4.9 (−43.0, 58.6)	0.85	16.5 (−27.7, 87.8)	0.53
Q3 (0.89–0.93)	142	34.9 (2.57, 77.5)	0.032	19.9 (−31.2, 109.3)	0.52	−11.0 (−47.4, 50.4)	0.66	28.4 (−18.2, 101.7)	0.28
Q4 (>0.93)	136	41.8 (5.81, 89.9)	0.019	−1.3 (−45.5, 78.8)	0.97	−30.2 (−60.1, 22.1)	0.21	15.5 (−26.1, 80.8)	0.53
* P*-trend		0.023		0.95		0.21		0.47	

^a^Linear model adjusting for race, menopausal status, history of diabetes, and molecular subtype.

**Table 4:** Associations between body composition and the mTOR pathway activities.

		mTOR		p-mTOR		Normalized p-mTOR		Total phosphoprotein	
		Percent difference (95% CI)^a^	*P*-value	Percent difference (95% CI)^a^	*P*-value	Percent difference (95% CI)^a^	*P*-value	Percent difference (95% CI)^a^	*P*-value
Fat mass, kg									
Q1 (≤24.25)	143	Ref.		Ref.		Ref.		Ref.	
Q2 (24.26–31.75)	136	−18.4 (−38.1, 7.5)	0.15	28.8 (−25.3, 120.0)	0.36	57.6 (−5.5, 162.8)	0.08	−28.2 (−54.7, 13.9)	0.16
Q3 (31.76–42.00)	139	4.8 (−20.8, 38.7)	0.74	38.6 (−20.3, 141.0)	0.25	32.3 (−21.3, 122.3)	0.29	−38.4 (−60.9, −2.9)	0.037
Q4 (>42.00)	133	11.0 (−16.8, 48.0)	0.48	149.8 (41.6, 341.0)	0.002	125.3 (32.2, 284.1)	0.003	−21.4 (−49.7, 23.0)	0.29
* P*-trend		0.24		0.002		0.009		0.10	
Fat mass index, kg/m^**2**^									
Q1 (≤9.19)	142	Ref.		Ref.		Ref.		Ref.	
Q2 (9.20–12.17)	137	−25.9 (−43.8, −2.3)	0.033	2.9 (−40.5, 78.1)	0.92	38.7 (−17.2, 132.3)	0.21	−16.8 (−47.6, 32.1)	0.43
Q3 (12.18–15.89)	138	10.2 (−16.8, 46.2)	0.50	25.3 (−28.4, 119.3)	0.43	13.7 (−32.8, 92.5)	0.63	−44.3 (−64.5, −12.5)	0.011
Q4 (>15.89)	134	12.6 (−15.5, 50.2)	0.42	124.1 (26.7, 296.4)	0.006	99.0 (16.4, 240.3)	0.012	−18.3 (−47.7, 27.8)	0.38
* P*-trend		0.11		0.005		0.032		0.21	
Percent body fat, %									
Q1 (≤35.3)	144	Ref		Ref.		Ref.		Ref.	
Q2 (35.4–40.5)	140	−32.6 (−48.9, −11.2)	0.005	38.1 (−20.4, 139.6)	0.25	104.3 (22.2, 241.7)	0.007	−13.4 (−45.6, 37.8)	0.54
Q3 (40.6–45.4)	134	−2.0 (−26.2, 30.1)	0.88	25.0 (−29.0, 120.1)	0.43	27.5 (−24.8, 116.1)	0.37	−34.1 (−58.0, 3.5)	0.07
Q4 (>45.4)	135	−7.2 (−30.4, 23.7)	0.61	123.9 (26.2, 297.0)	0.006	141.2 (41.3, 311.7)	0.001	−30.0 (−55.4, 9.9)	0.12
* P*-trend		0.74		0.012		0.013		0.50	

^a^Linear model adjusting for race, menopausal status, history of diabetes, and molecular subtype.

We examined the associations by ER status for BMI and percent body fat in relation to the expression of p-mTOR and normalized p-mTOR (Table 5). BMI ≥ 35 vs. <25 kg/m^2^ was associated with a higher level of normalized p-mTOR expression in ER− tumor (191.2%, 95% CI = 10.5%–667.9%, *P* = 0.031), but not in ER+ tumors. Similarly, Q4 vs. Q1 of percent body fat was associated with higher levels of p-mTOR and normalized p-mTOR expression in ER− tumors (207.6% and 283.3%, respectively, both *P* < 0.05), but not in ER+ tumors. These differences in associations between ER− and ER+ tumors were not statistically significant (*P*-heterogeneity >0.05).

**Table 5:** The expression of p-mTOR and normalized p-mTOR in ER+ and ER− tumors in relation to BMI and percent body fat.

	ER+ tumors		ER− tumors		*P*-heterogeneity^b^
	Percent difference^a^	*P*-value	Percent difference^a^	*P*-value	
BMI, kg/m^2^					
p-mTOR					0.75
<25	Ref.		Ref.		
25–29.99	−15.7 (−56.3, 62.5)	0.61	57.5 (−37.4, 296.1)	0.33	
30–34.99	19.7 (−38.4, 132.8)	0.60	7.1 (−61.4, 197.2)	0.89	
≥35	55.5 (−21.7, 208.9)	0.21	154.5 (−6.4, 591.7)	0.07	
* P*-trend	0.09		0.13		
Normalized p-mTOR					0.45
<25	Ref.		Ref.		
25–29.99	8.6 (−41, 100.9)	0.79	151.6 (2.8, 515.4)	0.043	
30–34.99	7.5 (−42.3, 100.5)	0.82	7.2 (−60.1, 188.3)	0.89	
≥35	39.4 (−26.7, 165.1)	0.31	191.2 (10.5, 667.9)	0.031	
* P*-trend	0.32		0.13		
Percent body fat, %					
p-mTOR					0.60
Q1 (≤35.3)	Ref.		Ref.		
Q2 (35.4–40.5)	−16.5 (57.3, 63.5)	0.60	94.8 (−24.2, 400.7)	0.17	
Q3 (40.6–45.4)	24.6 (−35.4, 140.2)	0.51	−14.3 (−69.8, 143.4)	0.77	
Q4 (>45.4)	49.7 (−24.1, 195.1)	0.24	207.6 (11.4, 749.0)	0.030	
* P*-trend	0.12		0.10		
Normalized p-mTOR					0.32
Q1 (≤35.3)	Ref.		Ref.		
Q2 (35.4–40.5)	38.9 (−25.7, 159.9)	0.30	169.5 (9.8, 561.8)	0.031	
Q3 (40.6–45.4)	30.4 (−29.3, 140.5)	0.39	−13.2 (−67.9, 134.3)	0.78	
Q4 (>45.4)	58.4 (−15.9, 198.4)	0.15	283.3 (45.9, 907.0)	0.007	
* P*-trend	0.21		0.049		

^a^Model adjusting for race, menopausal status, history of diabetes, and molecular subtype.

^b^*P*-value for the heterogeneity of the association between ER+ and ER− tumors; contrast tests.

## Discussion

In this study population comprised predominantly of Black women, we used a panel of IHC protein and phosphoproteins to indicate mTOR pathway activities. Among the assayed proteins, p-mTOR, both the original expression level and the normalized level, was associated with BMI and body fat mass. There was a pattern that the associations were more prominent in ER− than ER+ tumors, although the differences in associations between the subtypes were not significant.

Our study is among the few reporting the association between body fatness and mTOR pathway protein expression in breast tumors. Prior research reported obesity-related gene expression signatures in breast tumors^21,22^. Creighton et al.^22^ reported that gene expression of IGF-related proteins, which promote the mTOR pathway, was higher in obese verse normal weight breast cancer patients (*N* = 103). Fuentes-Mattei et al.^21^ observed significant upregulation of AKT/mTOR genes in obese vs. normal-weight women with ER+ tumors (*N* = 137). However, these findings were not replicated in a study of 519 postmenopausal women^23^. As obesity may affect both gene and protein expression in tumors, we focus on the latter because the mTOR pathway signaling is primarily through post-translational protein phosphorylation^24,25^. In our data, p-mTOR, but not p-AKT or p-p70S6K, in breast tumors was associated with body fatness, despite that one would expect these markers to yield similar results and they were modestly correlated. The discrepancies can be explained by the promotors of these proteins. mTOR is promoted by not only growth factors but also directly by nutrients and energy. AKT is activated by IGF1; however, IGF1 is not linearly associated with body fatness as the blood levels of IGF1 decreases when BMI ≥ 27 kg/m^2 ^^26^. Also, AKT signaling relies on the status of other proteins such as PI3K and PTEN. p70S6K can be signaled independently from mTOR, as it is downstream of phosphoinositide-dependent kinase-1 (PDK1) in the IGF signaling pathway^27^ and the ER signaling pathway^28^. Additional markers are needed to more comprehensively assess the singling pathways in breast cancer in relation to body fatness.

In the U.S., Black women with breast cancer have consistently lower survival than White women, despite a lower incidence of breast cancer until recent years^29,30^. In addition to societal and socioeconomic factors, such as access to screening and receipt of optimal treatment, tumor characteristics, e.g., grade, stage, and breast cancer subtype^31^, and molecular features^32–36^ likely contribute to differences in breast cancer mortality rates. We observed that the mTOR and p-mTOR expression were higher in Black women than White women after adjusting for tumor characteristics and BMI. The findings suggest that biological factors, such as race-related differences in insulin resistance^37^, may have a role in the racial disparity in the mTOR pathway activities and potentially survival. A limitation of our observation is that the sample size of White women was relatively small to Black women; the finding warrants replication.

In this study, mTOR pathway activities were higher in ER+ than ER− tumors. The finding is consistent with the biological evidence that there is a crosstalk between the ER and mTOR signaling pathways^38,39^. Nevertheless, it has been hypothesized that for ER− tumors, the influence of obesity may be more related to insulin signaling than estrogens^40,41^. Our observation on the associations of BMI and percent body fat with p-mTOR expression in ER− tumors supports the hypothesis. The sample size of women with ER− tumors was relatively small compared to ER+ tumors and the estimates had wide 95% confidence intervals. Thus, our findings in ER− tumors should be interpreted with caution and warrant confirmation. Clinically, it is important to know whether mTOR activation is related to the recurrence of these tumors in obese individuals and whether mTOR inhibition is helpful to prevent recurrence. mTOR inhibition can be achieved with mTOR inhibitors or metformin, an insulin sensitizer that is widely used in the treatment of type II diabetes^42,43^. Metformin reduces breast tumor growth in diet-induced obese mice^44^. In humans, metformin as adjuvant therapy with weight loss was found to be a safe strategy that modestly lowered circulating estrogen and insulin levels^45^. A large clinical trial (Canadian Cancer Trials Group’s MA.32) is ongoing to reveal the effect of metformin on breast cancer prognosis and survival^46^.

Other strengths of our study include rigorous and quantitative measurements of protein expression in breast tumors and body fatness. Immunostaining on FFPE tissue samples remains a cost-effective option for the assessment of protein expression in large epidemiologic studies, in which fresh-frozen tissue samples are unavailable. The stained tissue was manually annotated so that influences from other tissue components (e.g. stroma) on scoring were minimized. In the annotated images, using automated imaging analysis gives objective scores for each localization. Also, body size and body composition were measured by trained staff with a standardized protocol.

Limitations of our study should be noted. The study population was breast cancer cases from a case-control study, and body size and body composition might have been influenced by breast cancer development. In addition, body fatness might have been affected by breast cancer treatment because body fatness was measured within nine months after the time of breast cancer diagnosis and surgery. Research has shown a mean weight gain of 2 to 3 kg at 6 months to 1 year of chemotherapy in breast cancer patients^47,48^, while the weight gain is less for patients receiving surgery and radiation only^49^. Although we performed planned analyses with a priori hypotheses, the investigation of different measurements of body fatness resulted in multiple comparisons, potentially leading to false-positive results. Another limitation is that our 4-marker protein expression panel may have missed important signaling of the mTOR pathway activation. Future studies should measure additional proteins and mutations in the pathway for a more comprehensive assessment.

In conclusion, our study showed positive associations between body fatness and mTOR pathway activation, evident by a p-mTOR expression, in breast cancer. Our findings suggest that mTOR inhibition should be studied as a treatment strategy or as a method to prevent the recurrence of these tumors in obese individuals.

## Methods

### Study population

Study participants were breast cancer cases based on the Women’s Circle of Health Study (WCHS), a multi-site case-control study in New York City and New Jersey designed to investigate genetic and lifestyle risk factors for aggressive breast cancer. Details on study recruitment have been described elsewhere^50,51^. The protocol was approved by all relevant Institutional Review Boards. In brief, cases were self-identified Black and White women, 20–75 years of age, with no previous history of cancer other than non-melanoma skin cancer, and diagnosed within 9 months of ascertainment with primary, histologically confirmed, invasive breast cancer or ductal carcinoma in situ (DCIS). In-home interviews were conducted to obtain questionnaire data on known and suspected risk factors for breast cancer and anthropometric measurements. Of participants eligible for inclusion in this analysis, more than 95% signed a release form for their tumor tissue, as part of the informed consent. Formalin-fixed paraffin-embedded (FFPE) tissue specimens were used for tissue microarray (TMA) construction that was guided by a specialized breast pathologist (T.K.). TMAs included two to three tumor cores ranging in size from 0.6 to 1.2 mm. A total of 770 cases had tissue samples on a TMA for laboratory assays. After immunostaining, DCIS cases (*n* = 68) and cases that did not have any tumor tissue cores with sufficient cells (<25 cells) for scoring were excluded. The final data set consisted of 590 cases, including 464 black women (299 ER+ cases and 165 ER− cases) and 126 white women (97 ER+ cases and 29 ER− cases), who had data on all four assayed mTOR pathway proteins. Clinical and tumor characteristics, including the expression status of hormone receptors (HR, i.e., ER and progesterone receptor [PR]) and human epidermal growth factor receptor 2 (HER2), were obtained from the pathology reports.

### Ethics

All participants provided written informed consent to take part in the study. The protocol was approved by the Institutional Review Boards of all participating institutions, including Roswell Park Comprehensive Cancer Center and Rutgers Cancer Institute of New Jersey.

### Immunohistochemistry and image analysis

**Fig. 2:**
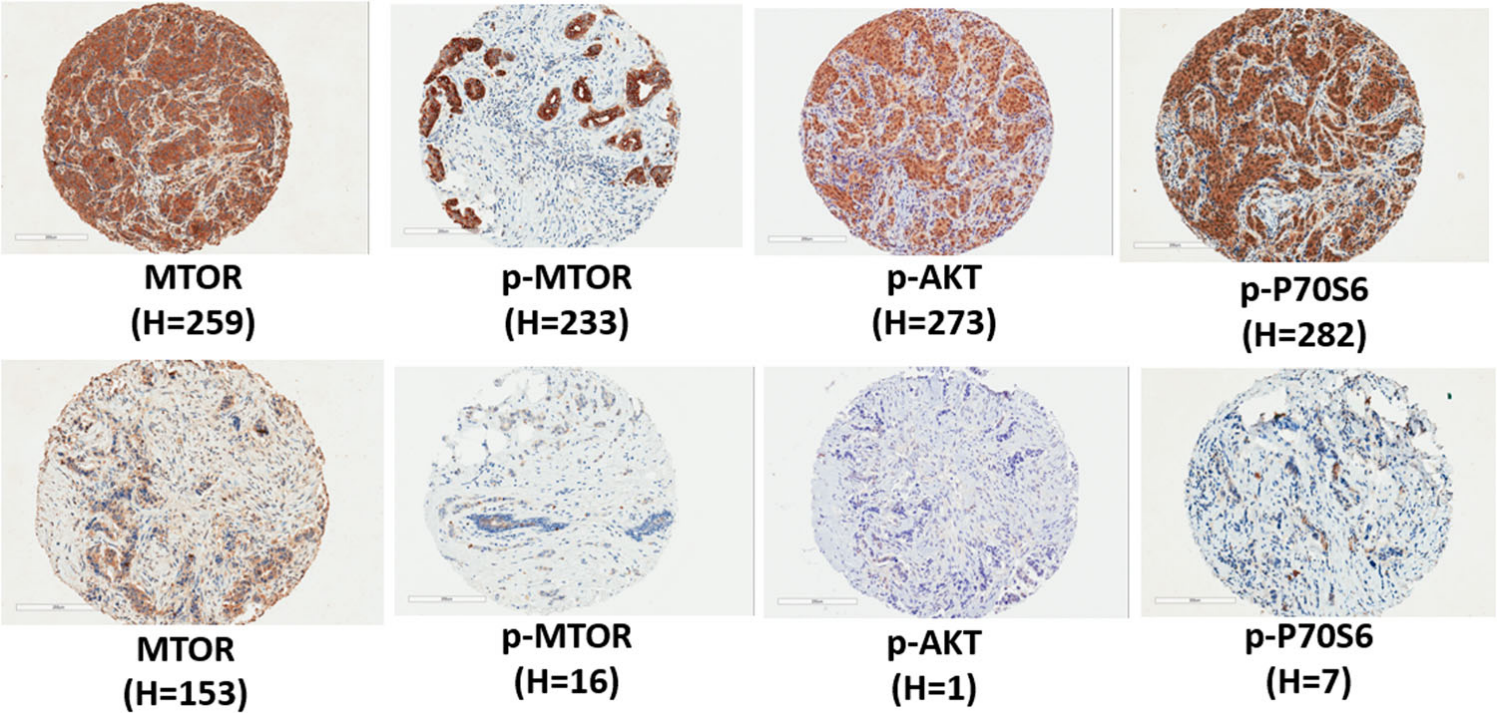
Representative images and *H*-scores of the mTOR IHC markers in breast tumor tissue. For each marker, a core with higher expression (top panel) and lower expression (bottom panel) was presented.

TMAs were sectioned at 5 µm and stained by immunohistochemistry (IHC) methods for mTOR (clone 7C10), phosphorylated mTOR (p-mTOR, Ser2448), phosphorylated AKT (p-AKT, Ser4731), and phosphorylated P70S6K (p-p70S6K, T389). The antibodies and IHC conditions for each stain are listed in Supplementary Table 5. Stained slides were digitally imaged at ×20 magnification using the Aperio ScanScope XT (Leica Biosystems), and the images were manually annotated to identify tumor for analysis. Automated image analysis on the annotated regions was performed using Aperio algorithms with minor adjustments for cell shape and intensity thresholds. The algorithms were validated by our study pathologist (W.B.) using positive and negative controls and tissue samples with different levels of intensity. Tumor cell cytoplasm was scored for mTOR and p-mTOR; cytoplasm and nuclei were scored for p-AKT and p-p70S6K. The percentage of cells stained was recorded in each intensity category: 0 (no staining), 1+ (only partial or weak staining), 2+ (moderate staining), and 3+ (strong staining). The core-level data were collapsed into patient-level data using a cellularity-weighted approach^52^. Core weight was defined as the number of tumor cells in a given core divided by the total number of tumor cells across all cores for that patient. The weighted average at the patient level was calculated by summing the product of percent positivity in each of the intensity category and core weight across all cores per patient. With the weighted average of percent positivity values, a histological score (*H*-score) at the patient level was calculated by the formula: [1 × (% cells 1+) + 2 × (% cells 2+) + 3 × (% cells 3+)] × 100^53^. Figure 2 show representative images of IHC staining.

### Anthropometric and body composition measurements

Anthropometric measurements were taken by trained staff during the in-person interviews using a standardized protocol described elsewhere^54^. Participants were asked to wear light clothing and to remove their shoes and any heavy jewelry. Waist and hip circumferences were measured by placing the measuring tape around the waist covering the umbilicus for waist and at the maximum extension of the buttocks in a horizontal plane for the hip. The waist and hip measurements were taken twice to the nearest 0.1 cm. If the difference between the first and second measurement was greater than 2 cm, a third measurement was taken. The two (or three) measurements were averaged for analyses. Standing height was measured once to the nearest 0.1 cm. Body composition was measured by bioelectrical impedance analysis using a Tanita® TBF-300A scale. Weight was measured once using the Tanita scale. BMI was classified as <25.0 (normal weight), 25.0–29.9 (overweight), 30.0–34.9 (class I obesity), and ≥35.0 kg/m^2^ (class II/III obesity)^55^. The fat mass index was calculated as a fat mass in kg divided by the square of height in meters.

### Statistical analysis

We examined the associations between each assayed protein and the body fatness measurements. In addition, because p-mTOR was stained for the specific phosphorylated site of mTOR and the levels of p-mTOR expression may depend on the expression of mTOR, we normalized the p-mTOR expression as the *H*-score of p-mTOR divided by the *H*-score of mTOR multiplied by 100. In addition, an *H*-score of total phosphoprotein was derived as the summation of *H*-scores from p-mTOR, p-AKT, and p-p70S6K to better represent the mTOR pathway activities than using single markers. Correlations of *H*-scores between individual proteins and between body fatness measurements were examined using the Pearson correlation coefficient. The protein expression levels were examined by demographic and clinicopathological characteristics using the analysis of variance (ANOVA). Linear regression was performed to assess the associations between the body fatness measurements, i.e., the independent variables and protein expression levels, i.e., the dependent variables. Because the distributions of *H*-scores were right-skewed, the data were log-transformed to improve the normality for the regression analysis. The regression coefficient (*β*) was converted to percentage using the formula: (exp (β) − 1) × 100% and interpreted as estimated percentage difference of protein expression for each category compared with the reference category. The BMI categories, as well as the quartiles of WC, WHR, fat mass, fat mass index, and percent body fat, were entered as categorical variables in regression models. Quartile 1 or BMI < 25 kg/m^2^ was chosen as the reference group. Other covariates were race (Black or White), menopausal status (premenopausal or postmenopausal), history of diabetes (ever or never), and breast cancer molecular subtype (HR+/HER2−, HR+/HER2+, HR−/HER2+, or HR−/HER2−)^56^. The effects of breast cancer stage and tumor grade were largely explained by molecular subtype and thus not included in the final model. Because the body size and composition measurements were highly correlated, only one measurement was included in a model to avoid multicollinearity. *P*-values for trend were obtained by treating the categories of body fatness measurement as continuous variables in the regression models. As planned, a stratification analysis was performed according to the ER status of breast cancer. To reduce the number of comparisons, only p-mTOR and normalized p-mTOR were examined for BMI and percent body fat in the stratification analysis, as the two proteins showed consistent results with BMI and the body fat variables in the main analysis. The difference in associations between ER+ and ER− tumors were examined using the contrast test method; p-values for heterogeneity were reported^57^. All tests of statistical significance were two-sided; a *P*-value < 0.05 was considered statistically significant. All analyses were planned and the results were not adjusted for multiplicity. Statistical analyses were performed using SAS, version 9.4 (SAS Institute Inc).

### Reporting summary

Further information on research design is available in the Nature Research Reporting Summary linked to this article.

## Supplementary information

SUPPLEMENTAL MATERIAL

Reporting Summary Checklist FLAT

## Data Availability

The data generated and analyzed during this study are described in the following metadata record: 10.6084/m9.figshare.12752582^58^. The data supporting the findings of this study are not publicly available in order to protect patient privacy. The data will be made available to authorized researchers who have submitted an IRB application. Please apply to the Women’s Circle of Health Study (WCHS) committee, email address: tingyuan.cheng@ufl.edu, for access to this data set.

## Code availability

Aperio (Leica Biosystems) was utilized in the automatic image analysis. The nuclear and cytoplasm algorithms can be available upon request.
