# Supplementary material for: Body fatness and mTOR pathway activation of breast cancer in the Women’s Circle of Health Study
Source: NPJ Breast Cancer. 2020 Sep 21;6:45. doi: 10.1038/s41523-020-00187-4 (PMC7505987; doi:10.1038/s41523-020-00187-4)
Supplement: Supplementary file 1 — SUPPLEMENTAL MATERIAL [file 41523_2020_187_MOESM1_ESM.pdf]

Supplementary Table 1. Pearson correlation coefficients of protein expression levels (log-transformed H-score)

	mTOR	p-mTOR	p-AKT	p-p70S6K	Normalized p-mTOR	Total phosphoprotein
mTOR	1.00	0.35 (<0.001)	0.28 (<0.001)	0.44 (<0.001)	0.13 (<0.002)	0.46 (<0.001)
p-mTOR	0.35 (<0.001)[1]	1.00	0.32 (<0.001)	0.27 (<0.001)	0.88 (<0.001)	0.71 (<0.001)
p-AKT	0.29 (<0.001)	0.32 (<0.001)	1.00	0.54 (<0.001)	0.20 (0.12)	0.68 (<0.001)
p-p70S6K	0.44 (<0.001)	0.27 (<0.001)	0.54 (<0.001)	1.00	0.07 (0.08)	0.70 (<0.001)
Normalized p-mTOR	-0.13 (<0.002)	0.88 (<0.001)	0.20 (0.12)	0.07 (0.08)	1.00	0.52 (<0.001)
Total phosphoprotein	0.46 (<0.001)	0.71 (<0.001)	0.68 (<0.001)	0.70 (<0.001)	0.52 (<0.001)	1.00

[1]P-values in parentheses

Supplementary Table 2. Association between race and mTOR pathway protein expression (N=562)

	**Black vs. White (Ref.)**	
**Protein**	**Percent difference (95% CI)[1]**	**P-value**
mTOR	71.1 (34.0, 118.4)	<0.001
p-mTOR	64.5 (1.6, 166.2)	0.043
Normalized p-mTOR	-3.8 (-39.2, 52.1)	0.87
Total phosphoprotein	13.5 (-23.2, 67.7)	0.53

[1] Model adjusting for age, menopausal status, history of diabetes, grade, stage, molecular subtype, and BMI

Supplementary Table 3. Pearson correlation coefficients of the body fatness measurements

	BMI	WC	WHR	Fat mass	Fat mass index	Percent body fat
BMI	1.00	0.88	0.34	0.93	0.97	0.80
WC	0.88	1.00	0.57	0.86	0.87	0.76
WHR	0.34	0.57	1.00	0.31	0.33	0.33
Fat mass	0.93	0.86	0.31	1.00	0.98	0.91
Fat mass index	0.97	0.87	0.33	0.98	1.00	0.92
Percent body fat	0.80	0.76	0.33	0.91	0.92	1.00

All $P<0.001$

Supplementary Table 4. Associations between body fatness measurements and the protein expression of p-AKT and p-p70S6K

		p-AKT		p-p70S6K	
	N	Percent difference (95% CI)[1]	P-value	Percent difference (95% CI)[1]	P-value
**BMI, kg/m²**					
<25	131	Ref.		Ref.	
25–29.99	164	-26.3 (-60.1, 35.9)	0.33	-55.7 (-76.2, -17.6)	0.010
30–34.99	146	18.5 (-37.0, 123.2)	0.60	-2.6 (-48.8, 84.9)	0.93
≥35	144	-18.1 (-57.1, 56.2)	0.54	-28.4 (-62.8, 37.9)	0.32
P-trend		0.97		0.97	
**WC, cm**					
Q1 (≤87.90)	151	Ref.		Ref.	
Q2 (87.91–98.40)	144	-16.1 (-54.2, 53.8)	0.57	-41.3 (-68.3, 8.8)	0.09
Q3 (98.41–110.00)	143	4.9 (-43.2, 93.7)	0.88	-22.0 (-58.3, 45.5)	0.43
Q4 (>110.00)	137	-0.6 (-47.8, 89.0)	0.98	-7.2 (-51.8, 78.5)	0.82
P-trend		0.84		0.98	
**WHR**					
Q1 (≤0.82)	150	Ref.		Ref.	
Q2 (0.83–0.88)	147	-7.9 (-49.8, 68.9)	0.79	15.4 (-37.9, 114.4)	0.65
Q3 (0.89–0.93)	142	2.2 (-45.1, 90.4)	0.94	0.2 (-46.9, 89.1)	0.99
Q4 (>0.93)	136	21.3 (-37.5, 135.6)	0.57	13.7 (-42.2, 123.9)	0.71
P-trend		0.53		0.84	
**Fat mass, kg**					
Q1 (≤24.25)	143	Ref.		Ref.	
Q2 (24.26–31.75)	136	-18.9 (-56.0, 49.6)	0.50	-42.8 (-69.6, 7.4)	0.08
Q3 (31.76–42.00)	139	8.9 (-41.5, 102.7)	0.79	-7.5 (-51.2, 75.5)	0.81
Q4 (>42.00)	133	-0.1 (-47.2, 89.2)	0.99	-17.9 (-57.5, 58.4)	0.56
P-trend		0.78		0.90	
**Fat mass index, kg/m²**					
Q1 (≤9.19)	142	Ref.		Ref.	
Q2 (9.20–12.17)	137	-21.4 (-57.6, 45.5)	0.44	-56.4 (-76.8, -17.9)	0.010
Q3 (12.18–15.89)	138	4.5 (-44.3, 95.9)	0.89	-22.6 (-59.4, 47.5)	0.44
Q4 (>15.89)	134	2.7 (-45.9, 94.9)	0.93	-25.9 (-61.6, 43.0)	0.37
P-trend		0.72		0.76	
**Percent body fat, %**					
Q1 (≤35.3)	144	Ref.		Ref.	
Q2 (35.4–40.5)	140	-12.7 (-52.9, 61.9)	0.67	-48.9 (-72.9, -3.8)	0.037
Q3 (40.6–45.4)	134	-25.7 (-60.6, 40.0)	0.36	-30.6 (-63.8, 33.0)	0.27
Q4 (>45.4)	135	11.4 (-41.4, 111.6)	0.74	-35.2 (-66.5, 25.3)	0.20
P-trend		0.86		0.36	

[1] Linear model adjusting for race, menopausal status, history of diabetes, and molecular subtype.

Supplementary Table 5. Immunohistochemistry antibodies and laboratory conditions

**Primary antibody**	**Clone**	**Vendor**	**Catalog No.**	**Antigen retrieval**	**Primary antibody dilution**	**Incubation duration**	**Secondary system**
mTOR (7C10)	7C10	Cell Signaling	2983	Flex TRS High pH (Agilent; catalog #GV80411), 30 mins	1/75	40 mins	Envision FLEX/HRP (Agilent; catalog #DM842)
p-mTOR (Ser2448)	49F9	Cell Signaling	2976	Flex TRS High pH, 30 mins	1/75	40 mins	Envision FLEX/HRP,
p-AKT (Ser473)	736E11	Cell Signaling	3787	Steamer 60 min in citrate buffer (PH=6)	1/22	16 hrs.	Rabbit Envision/ labeled polymer HRP anti -rabbit (Agilent; catalog #K4003)
p-p70S6K (T389)	E175 (ab32359)	Epitomics	1175-1	Flex TRS High pH, 30 mins	1/50	40 mins	Flex Rabbit Linker (Agilent; catalog# GV80911-2) was applied for 10 minutes followed by Flex /HRP polymer
