# Supplementary material for: Body fatness and mTOR pathway activation of breast cancer in the Women’s Circle of Health Study
Source: NPJ Breast Cancer. 2020 Sep 21;6:45. doi: 10.1038/s41523-020-00187-4 (PMC7505987; doi:10.1038/s41523-020-00187-4)
Supplement: Supplementary file 2 — Reporting Summary Checklist FLAT [file 41523_2020_187_MOESM2_ESM.pdf]

nature research

Corresponding author(s): Ting-Yuan David Cheng

Last updated by author(s): Mar 23, 2020

Reporting Summary

Nature Research wishes to improve the reproducibility of the work that we publish. This form provides structure for consistency and transparency in reporting. For further information on Nature Research policies, see Authors & Referees and the Editorial Policy Checklist.

Statistics

For all statistical analyses, confirm that the following items are present in the figure legend, table legend, main text, or Methods section.

n/a	Confirmed	
☐	☒	The exact sample size (n) for each experimental group/condition, given as a discrete number and unit of measurement
☐	☒	A statement on whether measurements were taken from distinct samples or whether the same sample was measured repeatedly
☐	☒	The statistical test(s) used AND whether they are one- or two-sided<br>*Only common tests should be described solely by name; describe more complex techniques in the Methods section.*
☒	☐	A description of all covariates tested
☐	☒	A description of any assumptions or corrections, such as tests of normality and adjustment for multiple comparisons
☐	☒	A full description of the statistical parameters including central tendency (e.g. means) or other basic estimates (e.g. regression coefficient) AND variation (e.g. standard deviation) or associated estimates of uncertainty (e.g. confidence intervals)
☐	☒	For null hypothesis testing, the test statistic (e.g. F, t, r) with confidence intervals, effect sizes, degrees of freedom and P value noted<br>*Give P values as exact values whenever suitable.*
☒	☐	For Bayesian analysis, information on the choice of priors and Markov chain Monte Carlo settings
☒	☐	For hierarchical and complex designs, identification of the appropriate level for tests and full reporting of outcomes
☐	☒	Estimates of effect sizes (e.g. Cohen's d, Pearson's r), indicating how they were calculated

Our web collection on statistics for biologists contains articles on many of the points above.

Software and code

Policy information about availability of computer code

Data collection	Aperio (Leica Biosystems) was utilized in the automatic image analysis. The nuclear and cytoplasm algorithms can be available upon request.
Data analysis	All data analysis performed using SAS version 9.4 as described in Methods.

For manuscripts utilizing custom algorithms or software that are central to the research but not yet described in published literature, software must be made available to editors/reviewers. We strongly encourage code deposition in a community repository (e.g. GitHub). See the Nature Research guidelines for submitting code & software for further information.

Data

Policy information about availability of data

All manuscripts must include a data availability statement. This statement should provide the following information, where applicable:

- Accession codes, unique identifiers, or web links for publicly available datasets
- A list of figures that have associated raw data
- A description of any restrictions on data availability

The datasets generated during and analyzed during the current study are not publicly available to protect patient privacy but are available from the corresponding author upon Institutional Review Board approval and reasonable request.

Field-specific reporting

Please select the one below that is the best fit for your research. If you are not sure, read the appropriate sections before making your selection.

☒ Life sciences ☐ Behavioural & social sciences ☐ Ecological, evolutionary & environmental sciences

For a reference copy of the document with all sections, see nature.com/documents/nr-reporting-summary-flat.pdf

Life sciences study design

All studies must disclose on these points even when the disclosure is negative.

Sample size	The sample size was based on the participants with tissue sample available in the WCHS study.
Data exclusions	DCIS cases (n=68) and cases that did not have any tumor tissue cores with sufficient cells (<25 cells) for scoring were excluded. DCIS were excluded because they are not considered invasive cancer. The criteria were pre-established.
Replication	Our quality control protocols were in place for body size and tumor protein expression measurements, as stated in Methods.
Randomization	Not applicable. This is a observational study.
Blinding	Not applicable. This is a observational study.

Reporting for specific materials, systems and methods

We require information from authors about some types of materials, experimental systems and methods used in many studies. Here, indicate whether each material, system or method listed is relevant to your study. If you are not sure if a list item applies to your research, read the appropriate section before selecting a response.

Materials & experimental systems

n/a	Involved in the study	
☐	☒	Antibodies
☒	☐	Eukaryotic cell lines
☒	☐	Palaeontology
☒	☐	Animals and other organisms
☐	☒	Human research participants
☒	☐	Clinical data

Methods

n/a	Involved in the study	
☒	☐	ChIP-seq
☒	☐	Flow cytometry
☒	☐	MRI-based neuroimaging

Antibodies

Antibodies used	We have provided the information in Supplemental Table 1.
Validation	All antibodies were previously validated by the manufactures.

Human research participants

Policy information about studies involving human research participants

Population characteristics	Study participants were breast cancer cases based on the Women's Circle of Health Study (WCHS), a multi-site case-control study in New York City and New Jersey. Cases were self-identified Black and White women, 20-75 years of age, with no previous history of cancer other than non-melanoma skin cancer, and diagnosed within nine months of ascertainment with primary, histologically-confirmed, invasive breast cancer or ductal carcinoma in situ (DCIS).
Recruitment	In NYC, cases were identified through collaborations with hospitals in Manhattan, Bronx, Brooklyn, and Queens with the largest referral patterns for AA women. In New Jersey, cases were identified through the NJ State Cancer Registry (NJSCR), using rapid case ascertainment in seven counties (Bergen, Essex, Hudson, Mercer, Middlesex, Passaic, and Union), with extension Monmouth and Burlington counties in 2012.
Ethics oversight	The protocol was approved by all relevant Institutional Review Boards, including Roswell Park Comprehensive Cancer Center and Rutgers Center Institute of New Jersey.

Note that full information on the approval of the study protocol must also be provided in the manuscript.
